# Evaluation of the optical and magnetic properties of novel Nd_0.9_Zn_0.1_FeO_3_ perovskite nanoparticles and their adsorption of Pb^2+^ ions from water

**DOI:** 10.1038/s41598-024-64936-2

**Published:** 2024-07-02

**Authors:** M. M. Arman

**Affiliations:** Materials Science Lab (1), Physics Department, Faculty of Science, Cairo University, Giza, Egypt

**Keywords:** Magnetic behavior, Optical properties, Perovskite, Nd_0.90_Zn_0.10_FeO_3_, The removal efficiency, Materials science, Physics

## Abstract

Nd_0.9_Zn_0.1_FeO_3_ was prepared in a single-phase with an average crystallite size of 25.82 nm using a citrate combustion technique. The energy dispersive X-ray assures the chemical formula of the sample. The elemental mapping of Zn-doped NdFeO_3_ illustrates the good homogeneous distribution of the elements in the sample. Nd_0.9_Zn_0.1_FeO_3_ has antiferromagnetic properties with weak ferromagnetic components and has good UV absorbance. The values of the band gap for the direct and indirect transitions are 1.473 eV and 1.250 eV, respectively. The adsorption of nickel(II), cobalt(II), chrome(VI), cadmium(II), and lead(II) ions has been studied at pH 7. The highest removal efficiency (η = 73.72%) was observed for the lead ions from water. The current study has examined the kinetics, recoveries, and mechanisms of utilizing Nd_0.90_Zn_0.10_FeO_3_ to remove Pb^2+^ ions from water. The optimum conditions for the absorbing Pb^2+^ are pH 7 and a contact time of 60 min. The Freundlich isotherm model is the best model for the absorption of Pb^2+^ ions. While, the pseudo-second-order kinetic model describes the kinetic adsorption data. The sample has a good efficiency for removing Pb^2+^ ions from water several times.

## Introduction

Drinking water resources are decreased by industrial contaminants and unreasonable exploitation of natural waterways^1^. The textile, refining, metallurgy, chemical, cosmetic, and agricultural sectors are the main polluters of natural waters^2^. As a result of the manufacturing of paints, galvanic coatings, and batteries, significant amounts of HMs are released into the aquatic environment. Heavy metals that are toxic are frequent pollutants^3,4^. Heavy metals can even cause cancer and induce neurological disorders^5^.

There are many effective methods to remove HMs from water, such as membrane filtration^6^, biological methods^7^, photocatalysis^8^, electrolysis^9^, and flocculation^10^. Among the other HMs removal methods, the adsorption method is the most efficient method for eliminating HMs due to its simplicity, high effectiveness, regeneration, and no slug. In the adsorption method, the HMs are redistributed from water to the adsorbent materials according to chemical or physical mechanisms.

The adsorption process is affected by many parameters, such as contact time, the surface area of the nanoparticles, the porosity of the samples, the pH value, and the function groups on the surface of the adsorbent materials. The good adsorbent materials of heavy metals are characterized by their thermal stability, such as zeolites^11^, graphene^12^, activated carbon^13^, zero-valent iron^14^, agricultural waste^15^, magnetic materials^16,17^, ZnO^18^, chitosan derivatives^19–22^, BaTi_0.8_Zr_0.2_O_3_^23^, and pectin‑ based biohydrogels^24^. Also, ferrite materials have received great attention from scientists for the removal of HMs from water^25,26^ due to their properties, such as being easy to prepare, thermal stability, and magnetic properties, which allow them to be extracted from the water by an external magnet and regenerated another time.

The orthoferrites have the ABO_3_ formula, where A is a large ionic radius cation such as rare earth elements, B is an ion with a small ionic radius, like transition metals, and O is the oxygen ions. Generally, orthoferrites AFeO_3_ have a perovskite crystal structure in which the A cation has 12 coordination numbers and the iron ions are coordinated by six oxygen ions, forming a FeO_6_ octahedron. There are many preparation techniques for prepared orthoferrites, such as solid-state reactions, coprecipitation, and the flash method. Among the prepared methods, the citrate combustion technique is inexpensive, effective, easy to prepare the nanoparticles, and fast^27^.

In the present work, Nd_0.9_Zn_0.1_FeO_3_ was synthesized using a combustion method and was characterized by XRD, FESEM, EDX, and elemental mapping. The physical properties of the Zn-doped NdFeO_3_ have been studied, such as their magnetic and optical properties. The ability of the sample to remove HMs from water was examined. Many parameters that affect the adsorption process of HM have been studied, such as the pH value, temperature, contact time, and regeneration of the material several times to remove the HM from water. The kinetic and isothermal models of adsorption have been studied.

## Experimental work

### Materials

Nd_0.9_Zn_0.1_FeO_3_ was prepared using a citrate combustion technique. The chemicals used in the preparation techniques are Zn nitrate, Nd nitrate, citric acid and Fe nitrate with high purity (99.9%). All chemicals were purchased from Sigma Aldrich. All the chemicals are of analytical quality, and they are used just as received, requiring no further purification.

### Preparation technique

Zn-doped NdFeO_3_ nanoparticles were synthesized using a citrate combustion method^16,28^. The metal nitrates with a stoichiometric ratio were dissolved in 25 ml of distilled water. A citric acid was added to the metal solution, where the citric acid: metal nitrate ratio is 1:1. Ammonia was used to bring the solution's pH to 7. The resultant solution was stirred and heated on the magnetic stirrer for one hour at 100 °C at a rate of 220 rpm, then heated for four hours at 250 °C until the solution converted to ash powder. A gate mortar was used to grind the ash powder for different characterizations and measurements.

### Characterization and measurements

XRD was used to study the crystallinity of the sample using a Bruker Advance D8 diffractometer. The morphology of the sample was investigated using FESEM (model Quanta 250), which was attached to EDX and elemental mapping. The magnetic hysteresis loop of the Zn-doped NdFeO_3_ has been investigated via VSM (VSM; 9600-1 LDJ, USA). The optical properties of Nd_0.9_Zn_0.1_FeO_3_ were investigated using a UV–visible spectrophotometer (Jasco V-630).

### HMs removal from water

To determine η of HMs such as nickel(II), cobalt(II), chrome(VI), cadmium(II), and lead(II) ions from water. 50 ppm standard solutions of HMs were prepared. Adding 10 ml of standard solutions in many beakers that contain 0.02 g of Zn-doped NdFeO_3_. Ammonia and diluted nitric acid were used to change the pH value. After shaking the solution on the electric shaker, 9 ml of the solution was withdrawn using a 0.2 µm syringe filter. Inductively coupled plasma spectrometry (ICP, Prodigy 7) was used to calculate the concentration of HMs.

## Results and discussion

**Figure 1:**
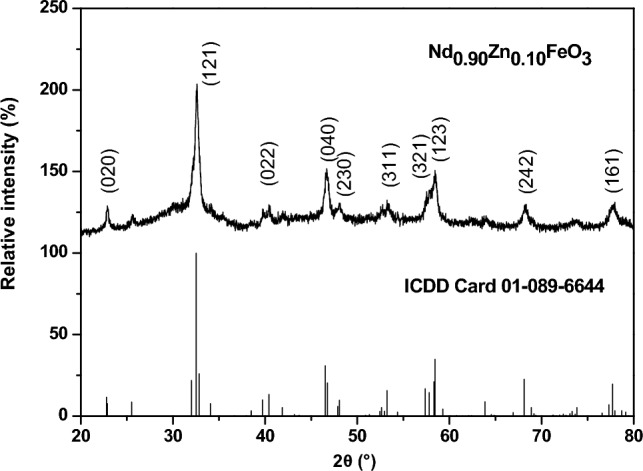
XRD of Nd_0.9_Zn_0.1_FeO_3_ and ICDD card number 01-089-6644.

Figure 1 illustrates the XRD of Nd_0.9_Zn_0.1_FeO_3_ nanoparticles. The zinc-doped NdFeO_3_ has an orthorhombic structure. The main peak at 2θ = 32.578° for (121) is characterized for the Nd_0.9_Zn_0.1_FeO_3_ perovskite. The noise in Fig. 1 indicates that the sample has a nanoscale. The average crystallite size was calculated using Scherer’s Eq. (1).

1 $$ L = \frac{0.94\lambda }{{\beta \cos \theta }} $$

where λ refers to the wavelength of the X-ray (λ = 1.5406 Å), θ is Bragg's angle, and β denotes the full width at half maximum. The average crystallite size (L) of Nd_0.9_Zn_0.1_FeO_3_ is 25.817 nm. Equation (2) was used to calculate the lattice parameters^29^.

2 $$ \frac{1}{{d^{2} }} = \frac{{h^{2} }}{{a^{2} }} + \frac{{k^{2} }}{{b^{2} }} + \frac{{l^{2} }}{{c^{2} }} $$

The lattice parameters (a, b, and c) were tabulated in Table 1. The values assure that Nd_0.9_Zn_0.1_FeO_3_ has an orthorhombic structure. The unit cell volume was calculated from Eq. (3).

3 $$ V = abc $$

**Table 1:** The lattice parameters, the unit cell volume, molecular weight, theoretical density, molecular weight, the tolerance factor, and the average crystallite size.

Sample	a (Å)	b (Å)	c (Å)	V (Å^3^)	M.W. (g/mol)	D_x_ (g/cm^3^)	L (nm)	t
Nd_0.90_Zn_0.10_FeO_3_	5.5416	7.7767	5.4525	234.98	240.19	6.7888	25.82	0.8757

The theoretical density (D_x_) of Nd_0.9_Zn_0.1_FeO_3_ nanoparticles was calculated from Eq. (4).

4 $$ D_{x} = \frac{ZM}{{NV}} $$

where M denotes the sample^’^s molecular weight, Z = 4, and N refers to Avogadro's number.

The tolerance factor (t) refers to the stability of the perovskite structure and gives the relation between the ionic radii of the A (r_A_), Fe (r_Fe_), and oxygen ions (r_O_), as shown in Eq. (5).

5 $$ {\text{t}} = \frac{{{\text{r}}_{{\text{A}}} + {\text{r}}_{{\text{O}}} }}{{\sqrt 2 \left( {{\text{r}}_{{{\text{Fe}}}} + {\text{r}}_{{\text{O}}} } \right)}} $$

The r_A_ value was estimated from Eq. (6).

6 $$ {\text{r}}_{{\text{A}}} = \, 0.{9}0r_{Nd}^{3 + } + 0.{1}0 r_{Zn}^{2 + } $$

The value of t is 0.8757, which indicates the orthorhombic structure of Nd_0.9_Zn_0.1_FeO_3_.

**Figure 2:**
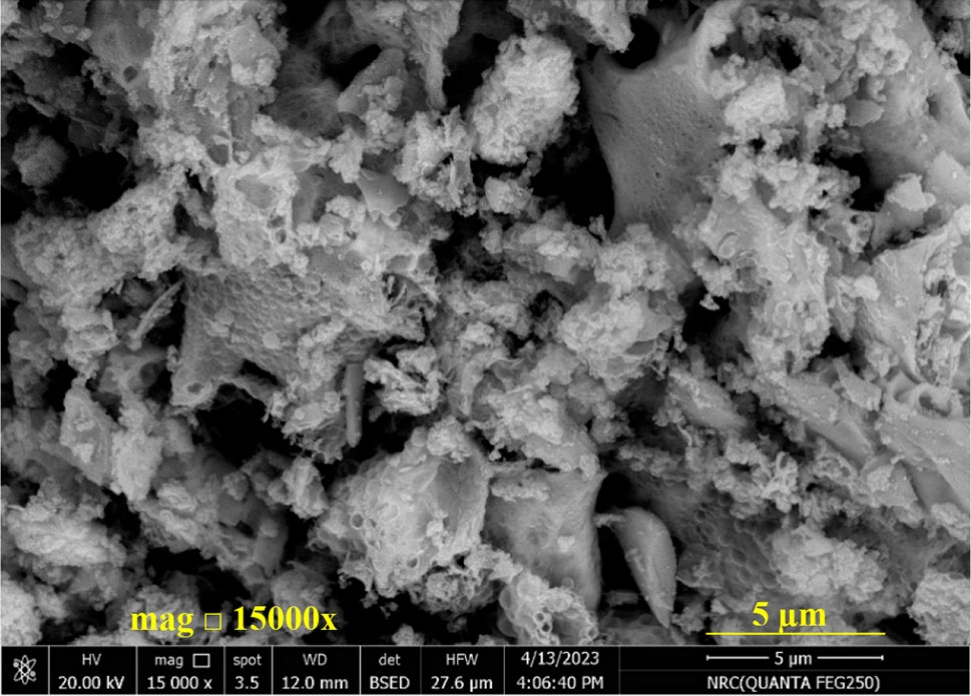
FESEM of the Nd_0.9_Zn_0.1_FeO_3_ nanoparticles.

**Figure 3:**
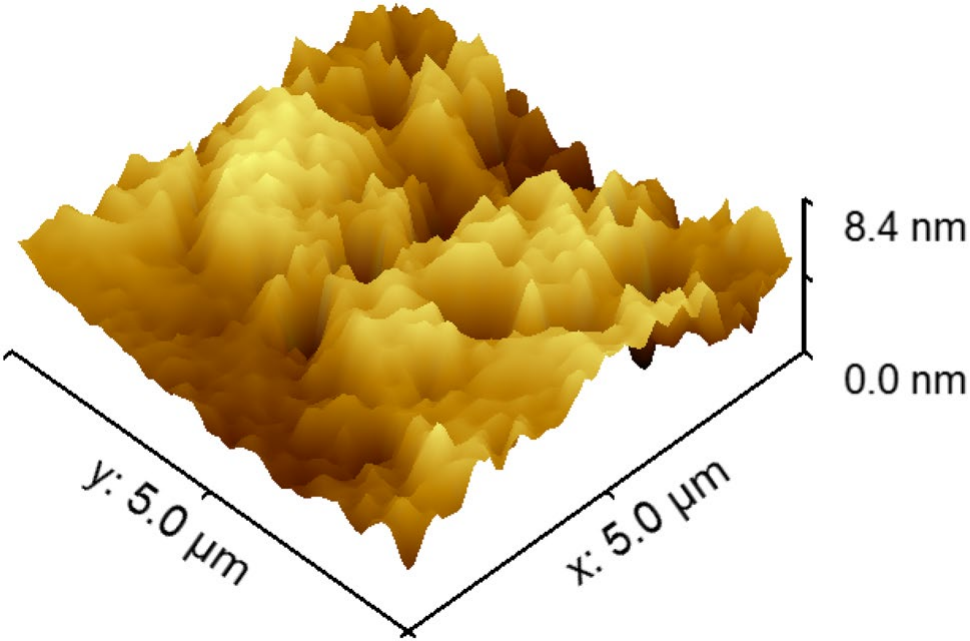
The roughness image of Nd_0.9_Zn_0.1_FeO_3_.

**Figure 4:**
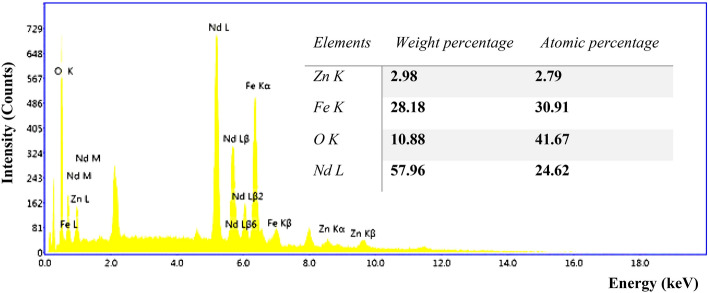
EDX of the Zn-doped NdFeO_3_. The inset table contains the weight and atomic percentages of the elements.

FESEM of the Nd_0.9_Zn_0.1_FeO_3_ nanoparticles, which have nanoscale Fig. 2 illustrates the agglomerated particles due to their magnetic properties and the preparation procedure^29^. The origin of the magnetic behavior of Nd_0.9_Zn_0.1_FeO_3_ is the antiparallel spines of Fe^3+^ ions. The FESEM image shows the particles have a porous and rough nature, which increased the surface area of Nd_0.9_Zn_0.1_FeO_3_ and increased its ability to adsorb the HMs. The roughness of the surface of Zn-doped NdFeO_3_ was studded using Gwyddion 2.50 software as illustrated from Fig. 3. The average roughness value and the maximum peak height are 5.09 nm and 3.30 nm, respectively. The presence of humps and grooves on the surface of the sample refers to the porous nature of the surface^30^. Figure 4 illustrates the EDX of the Nd_0.9_Zn_0.1_FeO_3_ nanoparticles. The figure shows the presence of Fe, Zn, O, and Nd elements without any impurities. The weight percent (wt%) and the atomic percent (wt%) of the elements were illustrated in the inset table, which assures the preparation of the sample in the same chemical formula: Nd_0.9_Zn_0.1_FeO_3_.

**Figure 5:**
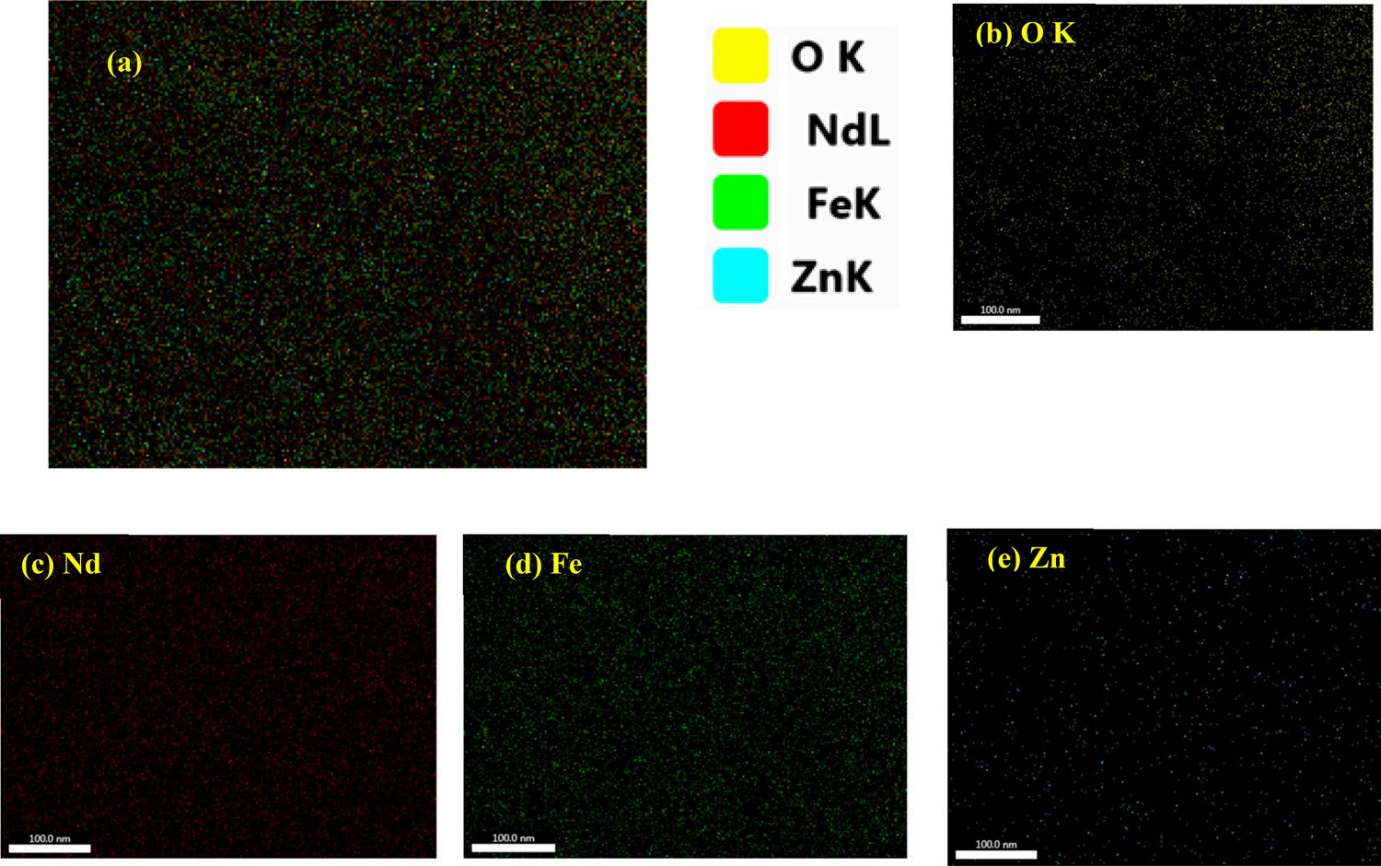
The elemental mapping of Zn-doped NdFeO_3_.

Figure 5 illustrates the elemental mapping of Zn-doped NdFeO_3_. The good homogeneous distribution of the elements is shown in Fig. 5a. Each element in the sample was illustrated by a specific color in Fig. 5b–e.

**Figure 6:**
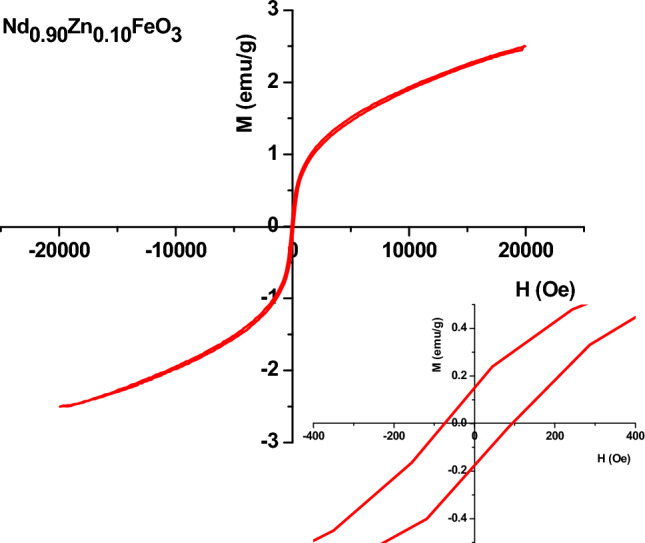
The magnetization curve of the Nd_0.9_Zn_0.1_FeO_3_.

Figure 6 illustrates the magnetization curve of the Nd_0.9_Zn_0.1_FeO_3_ nanoparticles. The M-H loop shows the s-shape hysteresis loop without saturation, which indicates the antiferromagnetic properties of the Zn-doped NdFeO_3_ nanoparticles. The magnetic interactions originate from the magnetic coupling between the Nd and Fe spins. The nanosized NdFeO_3_ has weak ferromagnetic properties, as reported in many works^31,32^. Zn^2+^ is a nonmagnetic ion because the electrons are aligned and paired in the valence orbitals. The magnetic behavior of Nd_0.9_Zn_0.1_FeO_3_ originates from^31,33^:

1. The superexchange interaction between Fe^3+^ ions via O^2−^ ions.
2. The direct interaction between Fe^3+^ ions.
3. The magnetic interaction between Nd^3+^–O^2−^–Nd^3+^ and Nd^3+^–O^2−^–Fe^3+^.

These magnetic interactions lead to the antiferromagnetic properties of the Zn-doped NdFeO_3_.

The values of M_r_, M_s_, and the coercive field (H_c_) are reported in Table 2. M_s_ illustrates the antiferromagnetic properties of the Zn-doped NdFeO_3_. The squareness ratio was calculated according to the following equation:

7 $$ SQR = \frac{{M_{r} }}{{M_{s} }} $$

**Table 2:** The saturation magnetization (M_s_), the remanence magnetization (M_r_), the exchange bias field, and the magnetic anisotropic constant (K).

Sample	M_s_ (emu/g)	M_r_ (emu/g)	H_c_ (Oe)	SQR = M_r_/M_s_	H_EB_ (Oe)	K (erg/g)
Nd_0.90_Zn_0.10_FeO_3_	2.5036	0.1629	84.085	0.065	− 9.845	219.287

The value of the SQR is 0.065, which indicates the magneto-static interactions between the particles.

The shift of the hysteresis loop to the positive direction is due to the exchange bias (EB) field^34^. The EB originates from the coupling between the ferromagnetic spins and AFM spins in the sample. H_EB_ was calculated from Eq. (8) and reported in Table 2.

8 $$ H_{EB} = \frac{{ - \left[ {H_{1} + H_{2} } \right]}}{2} $$

where H_1_ and H_2_ are the intercepts of the M-H loop with the negative and positive x-axes, respectively.

Based on the non-collinearity of the spins at the surface of the sample, magnetic anisotropy was present in the Zn-doped NdFeO_3_. Equation (9) was used to calculate the value of K.

9 $$ K = \frac{{H_{C} \times M_{S} }}{0.96} $$

**Figure 7:**
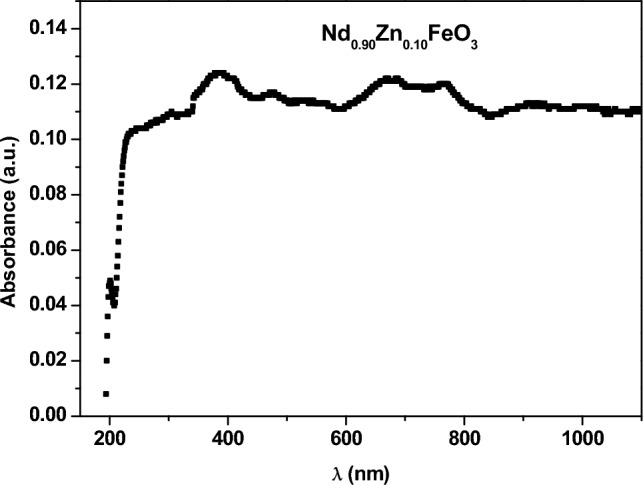
The dependence of the absorbance on the photon wavelength.

Figure 7 illustrates the dependence of the absorbance and the photon wavelength. The figure contains two regions, the first at the low wavelength (λ ≤ 370 nm) and the second at the high wavelength (λ > 370 nm). In the first region, λ is small and photon energy is high, which enables the electrons to transfer from the valence band (V.B.) to the conduction band (C.B.). In the second region, λ is high and the photon’s energy is low, and they cannot jump the electrons from V.B. to C.B.

**Figure 8:**
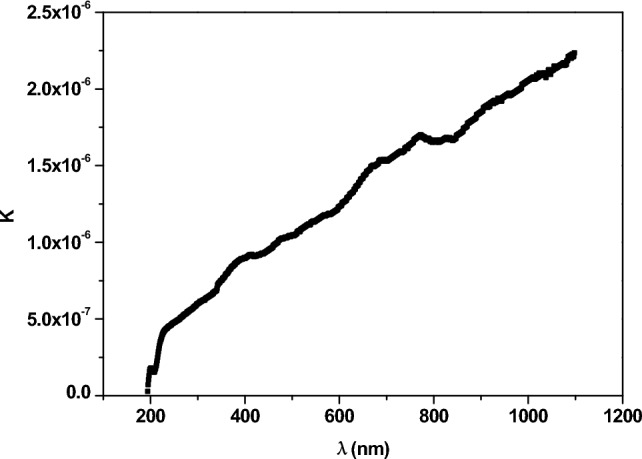
The relationship between the optical extinction coefficient and the wavelength of the spectra.

The optical extinction coefficient (k) is correlated with the electromagnetic energy loss in the sample. Equation (10) was used to calculate the values of k.

10 $$ k = \frac{\alpha \lambda }{{4\pi }} $$

where α is the optical absorption coefficient and can be calculated using the following equation:

11 $$ \alpha = \frac{{\left( {2.303} \right) A}}{l} $$

where l refers to the spaceman’s length and A is the absorbance. Figure 8 depicts how k depends on λ. The value of k increases with raising the wavelength of the photons, owing to the fact that by increasing the λ, the photon’s energy (hν) decreases and they cannot jump the electrons from V.B. to C.B., so the electromagnetic energy dissipates and k increases.

**Figure 9:**
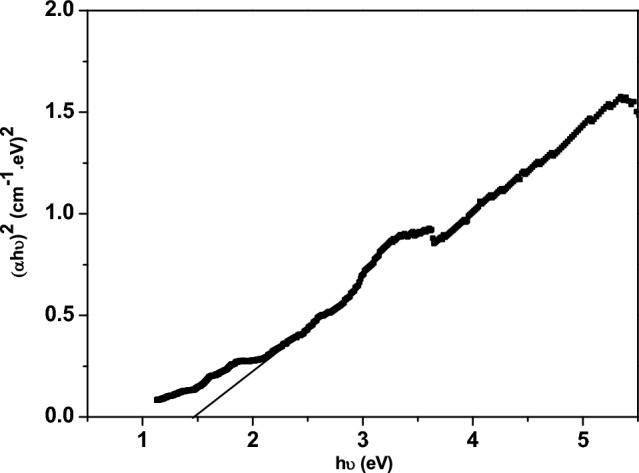
Direct transition Tauc plot for Zn-doped NdFeO_3_.

**Figure 10:**
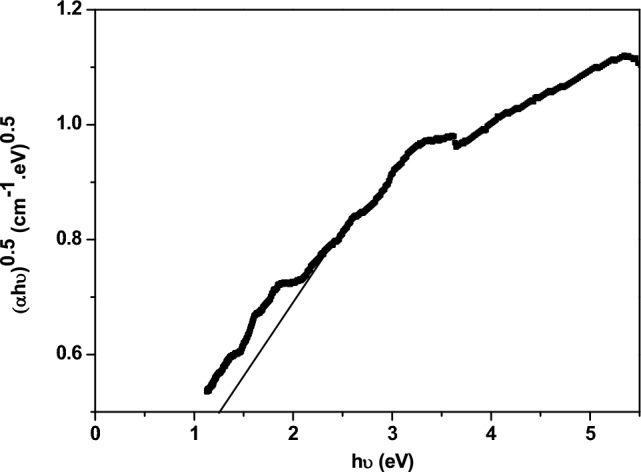
Indirect transition Tauc plot for Nd_0.90_Zn_0.10_FeO_3_.

The optical band gap (E_g_) of Zn-doped NdFeO_3_ can be determined using the Tauc plot. Equation (12) represents the Tauc equation^18,35^.

12 $$ (\alpha hv)^{x} = A\left( {hv - E_{g} } \right) $$

where A is a constant. The type of the optical transition can be estimated from the value of (x), where x = 2 for the direct transition and x = 0.5 for the indirect transition. Figure 9 shows the direct transition Tauc plot for the Zn-doped NdFeO_3_ nanoparticles, while Fig. 10 illustrates the indirect transition Tauc plot. The values of the direct and indirect energy gaps were reported in Table 3, which indicate that the sample can be used as a photocatalyst.

**Table 3:** The E_g_ values for direct and indirect transitions.

Sample	Direct band gap (E_g dir_) (eV)	Indirect band gap (E_g indir_) (eV)
Nd_0.90_Zn_0.10_FeO_3_	1.473	1.250

**Figure 11:**
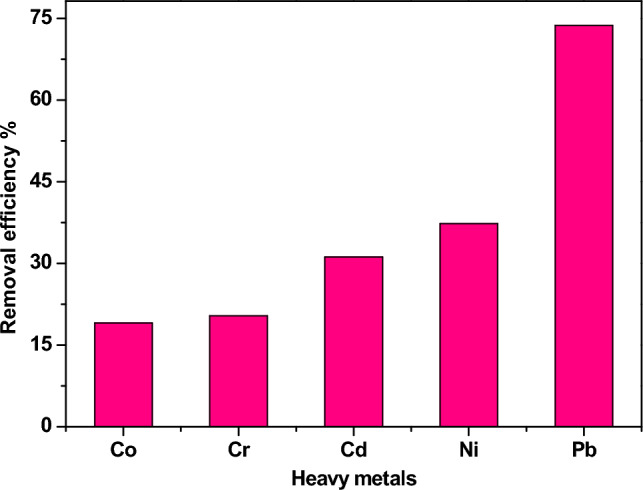
The η of different HMs using Zn-doped NdFeO_3_.

Figure 11 shows the relationship between η of the investigated sample and the type of heavy metals at pH 7. Equation (13) was used to calculate the value of the HM removal efficiency (η).

13 $$ \eta = \frac{{C_{i} - C_{f} }}{{C_{i} }} \times 100 $$

where $C_{f}$ denotes the final concentration while and $C_{i}$ refers to the initial concentration of the heavy metals. Nd_0.90_Zn_0.10_FeO_3_ nanoparticles have a good ability to adsorb many HMs from aqueous solutions. The highest removal efficiency (η = 73.72%) was observed for the Pb^2+^ ions from water. So studying the parameters that affect the absorption process will focus on the lead ions.

### ***Effect of pH on the adsorption of Pb***^***2***+^***from water***

**Figure 12:**
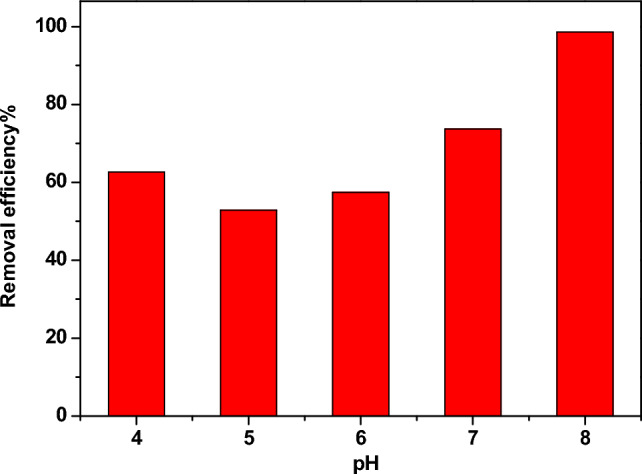
The dependence of η of Pb^2+^ ions from water on the pH value.

Figure 12 illustrates the dependence of η on the pH value. At low pH values (acidic medium), the adsorption of the Pb^2+^ ions is small due to the presence of excess H^+^ ions in the solution, which competes with the adsorption of the Pb^2+^ ions on the active cites. At high pH values (basic medium), the Pb^2+^ ions precipitate as lead hydroxides, which is not favorable^36^. At pH = 7, the Pb^2+^ ions are easily absorbed on the surface active sites of the investigated sample, so the optimum pH value for the adsorption of Pb^2+^ is 7.

### The influence of contact time

**Figure 13:**
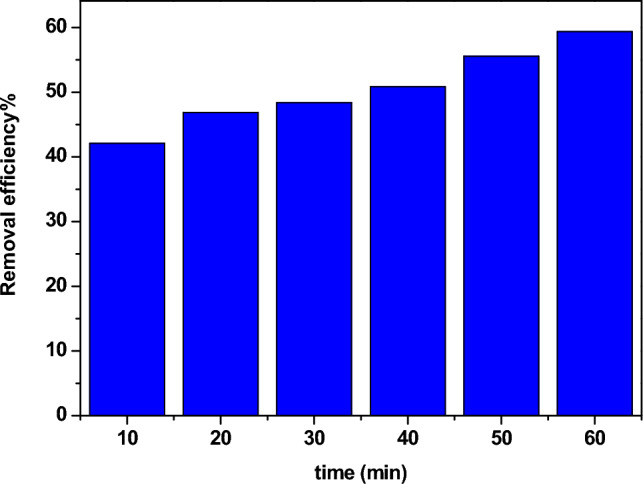
The influence of contact time on η of lead from water.

Figure 13 shows the dependence of the adsorption of Pb^2+^ ions on the contact time. It is illustrated that as the contact time increased, the adsorption increased. At the start of the experiment, the active site number was large, which could adsorb Pb^2+^ ions. While raising the contact time, the adsorption increases due to more lead ions being adsorbed on the investigated sample. From Fig. 13, the optimum contact time is observed at 60 min.

### Adsorption isotherm models

**Figure 14:**
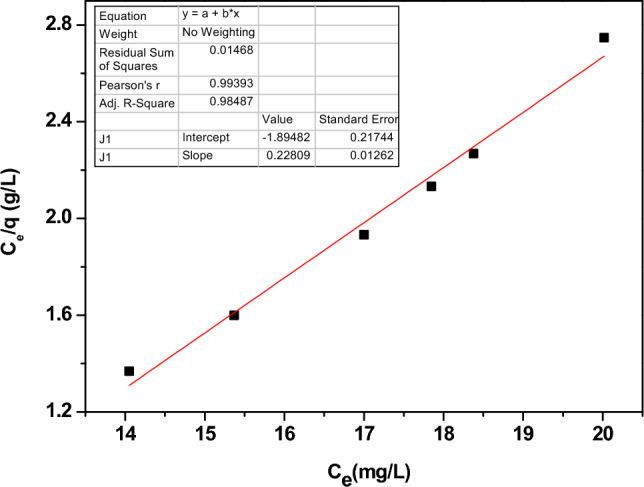
The relation between C_e_/q and C_e_ for Zn-doped NdFeO_3_.

The monolayer adsorption of Pb^2+^ removal onto an Zn-doped NdFeO_3_ surface is described by the Langmuir model. Since the surface has limited active sites and homogeneous adsorption energy, the Langmuir model states that once Pb^2+^ ions bind to a binding site, no further adsorption can take place. Equation (14) describes the Langmuir isotherm model.

14 $$ \frac{{C_{e} }}{{q_{e} }} = \frac{1}{{q_{m} K_{L} }} + \frac{{C_{e} }}{{q_{m} }} $$

where K_L_ is the Langmuir constant, C_e_ refers to the equilibrium metal concentration, q_m_ denotes the highest adsorption capacity, and q_e_ denotes the adsorption concentration. Figure 14 illustrates the Langmuir isotherm model for Zn-doped NdFeO_3_. The correlation coefficient (R^2^) of the Langmuir model is listed in Table 4.

**Table 4:** The Langmuir constant, R^2^, and the adsorption intensity for the Langmuir and Freundlich models.

Sample	Langmuir model		Freundlich model	
	R^2^	$\frac{1}{{q_{m} }}$ (mg/g)^−1^	R^2^	$\frac{1}{{\text{n}}}$
Nd_0.90_Zn_0.10_FeO_3_	0.9848	0.228	0.9851	− 0.955

The Freundlich isotherm, which is represented by Eq. (15), is used to study the multilayer adsorption of Pb^2+^ ions on heterogeneous surfaces.

15 $$ {\text{q}}_{{\text{e}}} = {\text{ K}}_{{\text{f}}} C_{e}^{\frac{1}{n}} $$

where $\frac{1}{n}$ denotes the adsorption intensity, and K_f_ refers to the Freundlich constant. By taking the logarithm for both sides of Eq. (15), the Freundlich model equation becomes Eq. (16).

16 $$ {\text{Ln}} q_{e} = {\text{Ln}} K_{f} + \frac{1}{n}{\text{Ln}} C_{e} $$

**Figure 15:**
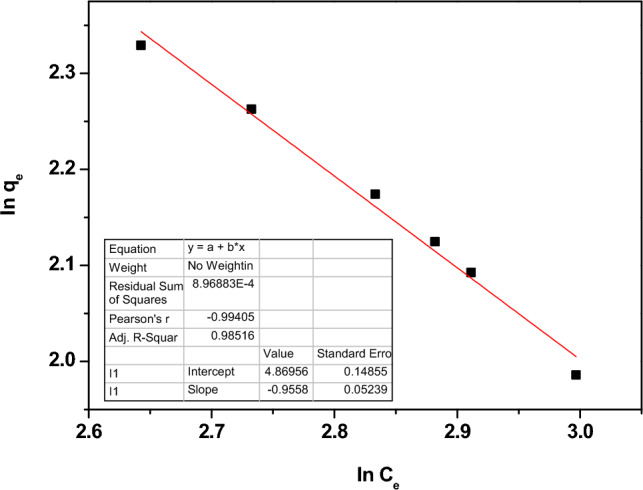
The relation between the lnq_e_ and the lnC_e_ for Nd_0.90_Zn_0.10_FeO_3_.

Figure 15 illustrates the Freundlich model for the investigated sample. Table 4 contains the values of R^2^.

From Table 4, the R^2^ value of the Freundlich model is greater than that of the Langmuir model, which means that the Freundlich model is the most fit model.

### The influence of temperature

**Figure 16:**
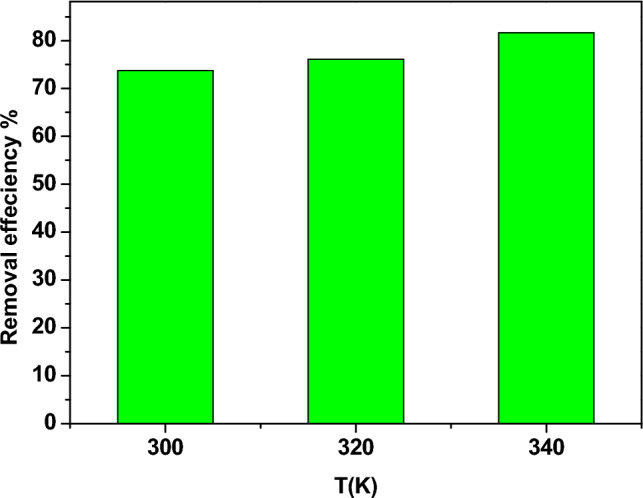
The dependence of the removal efficiency on the temperature.

Figure 16 illustrates the influence of temperature on the adsorption of Pb^2+^ on the surface of Zn-doped NdFeO_3_. The temperature has a small effect on η in the temperature range of 300–340 K. The η of lead ions rose with increasing temperature, which indicates that the adsorption process is not only a physical adsorption but also a chemical adsorption. The active site number on the adsorbent surface increases with increasing temperature due to the bond rupture^37,38^. In the presence study, the adsorption is multilayer and applies the Freundlich isotherm.

### Adsorption kinetic models

The kinetic models were used to study the type and rate of adsorption on Zn-doped NdFeO_3_. The pseudo-first-order, pseudo-second-order, and inter-particle diffusion kinetic models are represented by the following equations, respectively.

17 $$ \ln (q_{e} - q_{t} ) = \ln q_{e} - \frac{{k_{1} }}{2.303}t $$

18 $$ \frac{t}{{q_{t} }} = \frac{1}{{k_{2} q_{e}^{2} }} + \frac{t}{{q_{e} }} $$

19 $$ q_{t} = k_{3 } t^{\frac{1}{2}} + C $$

where q_t_ is the Pb^2+^ adsorbed at time t. The diffusion rate constants of pseudo-1st, 2nd, and inter-particles denote K_1_, K_2_, and K_3_, respectively.

**Figure 17:**
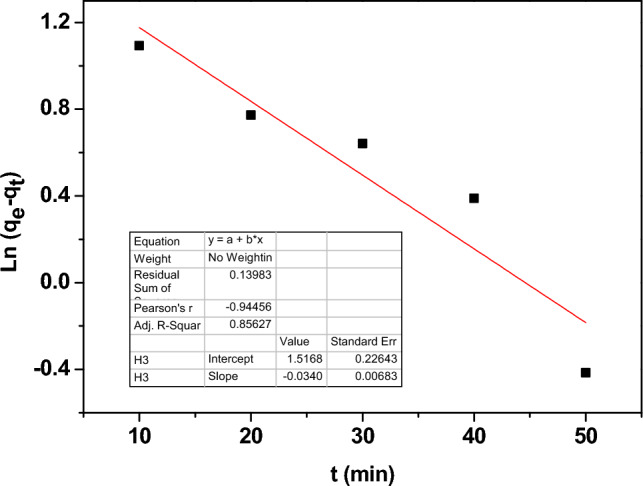
The pseudo-first order model for adsorption of Pb^2+^ from water.

**Figure 18:**
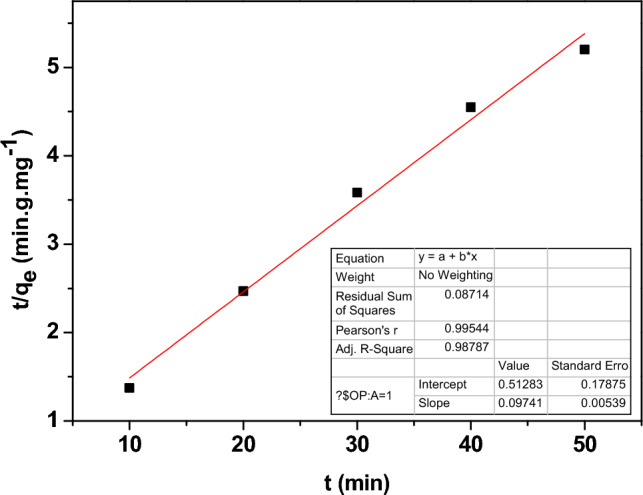
The pseudo-second-order model for adsorption of Pb^2+^ from water.

**Figure 19:**
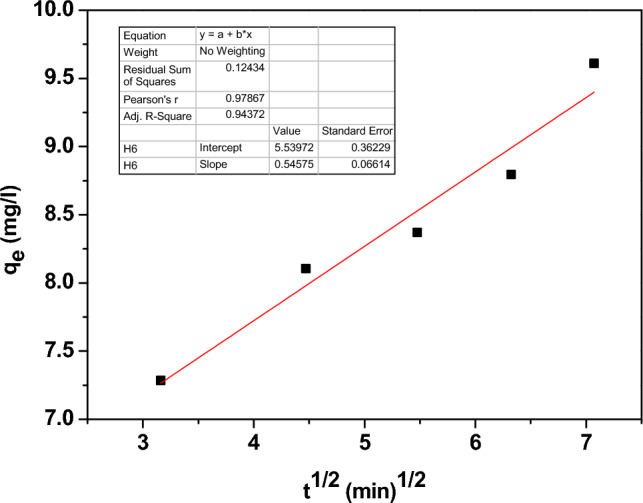
The inter-particle diffusion model of lead absorption from water.

Figure 17 illustrates the pseudo-first order, which describes the weak and reversible physisorption mechanism. While Fig. 18 represents the pseudo-second order and refers to the presence of a chemisorption mechanism between the Pb^2+^ and the surface active sites of Zn-doped NdFeO_3_, in the chemisorption mechanism, there are strong covalent bonds between the Pb^2+^ ions and the adsorbent. Contrarily, Fig. 19 shows the intra-particle diffusion model, which is a quick and well-thought-out process. The value of the regression coefficient R^2^ (> 0.98) is the maximum for the pseudo-second order, which means that the absorption is a chemisorption mechanism.

### Regeneration test

**Figure 20:**
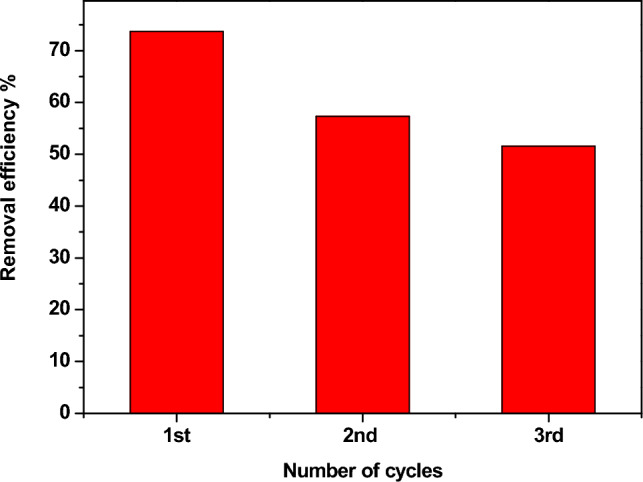
The regeneration of Nd_0.90_Zn_0.10_FeO_3_ for removing the lead ions from water several times.

To make the adsorption process more useful and less expensive, Zn-doped NdFeO_3_ nanoparticles were used to remove Pb^2+^ ions from water three times. Figure 20 illustrates the removal of Pb^2+^ ions for three cycles with high removal efficiency. The values of η are 73%, 57%, and 52% for the first, second, and third cycles.

## Conclusion

Nd_0.90_Zn_0.10_FeO_3_ perovskite was synthesized using a citrate combustion method and was characterized by XRD, EDX, elemental mapping, and FESEM. XRD reveals that Nd_0.90_Zn_0.10_FeO_3_ has a L of 25.817 nm. The FESEM image illustrates the porous nature of the prepared sample. Nd_0.9_Zn_0.1_FeO_3_ has antiferromagnetic behavior with a M_s_ of 2.5036 emu/g. Zn-doped NdFeO_3_ has a good ability to absorb UV and visible light with a small optical band gap. The presence of HMs in water is a great environmental problem, and the investigated sample has a good ability to remove them from water. The highest removal efficiency of Pb^2+^ ions from water at pH 7 is 73.72%. The pseudo-second-order kinetic model and the Freundlich isotherm model are the most fitting models for the experimental data. The absorption of Pb^2+^ on the surface of Nd_0.9_Zn_0.1_FeO_3_ is a chemisorption mechanism. The sample can be considered a good absorbing material for the removal of lead from water several times. In future work, the investigated sample will be used to prepare a nanocomposite with a metal oxide to increase the removal efficiency of heavy metals and dyes from water.

## Data Availability

Data of the current work are available from the corresponding author on reasonable request.

## References

[1] Reddy, D. H. K. Water pollution control technologies (2017).‏

[2] Vieira MLG, Esquerdo VM, Nobre LR, Dotto GL, Pinto LAA (2014). Glass beads coated with chitosan for the food azo dyes adsorption in a fixed bed column. J. Ind. Eng. Chem..

[3] Latif A, Sheng D, Sun K, Si Y, Azeem M, Abbas A, Bilal M (2020). Remediation of heavy metals polluted environment using Fe-based nanoparticles: Mechanisms, influencing factors, and environmental implications. Environ. Pollut..

[4] Naushad MJCEJ (2014). Surfactant assisted nano-composite cation exchanger: Development, characterization and applications for the removal of toxic Pb^2+^ from aqueous medium. Chem. Eng. J..

[5] Ahmad SZN, Salleh WNW, Ismail AF, Yusof N, Yusop MZM, Aziz F (2020). Adsorptive removal of heavy metal ions using graphene-based nanomaterials: Toxicity, roles of functional groups and mechanisms. Chemosphere.

[6] Aloulou W, Aloulou H, Khemakhem M, Duplay J, Daramola MO, Amar RB (2020). Synthesis and characterization of clay-based ultrafiltration membranes supported on natural zeolite for removal of heavy metals from wastewater. Environ. Technol. Innov..

[7] Wen Q, Wang Q, Li X, Chen Z, Tang Y, Zhang C (2018). Enhanced organics and Cu^2+^ removal in electroplating wastewater by bioaugmentation. Chemosphere.

[8] Kannan K, Radhika D, Nikolova MP, Andal V, Sadasivuni KK, Krishna LS (2020). Facile microwave-assisted synthesis of metal oxide CdO-CuO nanocomposite: Photocatalytic and antimicrobial enhancing properties. Optik.

[9] Gyliene O, Tarozaite R, Nivinskiene O (2004). Sorption of Ni (II)-citrate complex from electroless nickel plating solutions onto chitosan. Trans. IMF.

[10] Sun Y, Zhou S, Pan SY, Zhu S, Yu Y, Zheng H (2020). Performance evaluation and optimization of flocculation process for removing heavy metal. Chem. Eng. J..

[11] Belova, T. P. Adsorption of heavy metal ions (Cu^2+^, Ni^2+^, Co^2+^ and Fe^2+^) from aqueous solutions by natural zeolite. *Heliyon*, **5(9)** (2019).‏ 10.1016/j.heliyon.2019.e02320 PMC6731206 31517110

[12] Zhang K, Li H, Xu X, Yu H (2018). Synthesis of reduced graphene oxide/NiO nanocomposites for the removal of Cr (VI) from aqueous water by adsorption. Microporous Mesoporous Mater..

[13] Hu Z, Lei L, Li Y, Ni Y (2003). Chromium adsorption on high-performance activated carbons from aqueous solution. Sep. Purif. Technol..

[14] Wang J, Pang H, Tang H, Yu S, Zhu H, Wang X (2020). Carbothermic synthesis of carbon-supported zero-valent iron material for removal of U (VI) from aqueous solution. J. Inorg. Mater..

[15] Gupta S, Babu BV (2009). Removal of toxic metal Cr (VI) from aqueous solutions using sawdust as adsorbent: Equilibrium, kinetics and regeneration studies. Chem. Eng. J..

[16] Arman MM (2023). Novel multiferroic nanoparticles Sm_1−x_Ho_x_FeO_3_ as a heavy metal Cr^6+^ ion removal from water. Appl. Phys. A.

[17] Abdul-Raheim MAR, Shimaa MES, Reem KF, Manar EAR (2016). Low cost biosorbents based on modified starch iron oxide nanocomposites for selective removal of some heavy metals from aqueous solutions. Adv. Mater. Lett..

[18] Arman MM, Gamal AAR (2023). Role of Gd^3+^ and Ho^3+^ doping on the structure, physical properties and applications of ZnO. Appl. Phys. A.

[19] Abdel-Raouf MES, Farag RK, Farag AA, Keshawy M, Abdel-Aziz A, Hasan A (2023). Chitosan-based architectures as an effective approach for the removal of some toxic species from aqueous media. ACS Omega.

[20] Abdel-Raouf MES, Farag RK, Farag AA, Keshawy M, Abdel-Aziz A, Hasan A (2023). Optimization, kinetics, and isotherm studies of methyl thioninium chloride removal from simulated solutions using chitosan derivatives. ACS omega.

[21] El-Sayed Abdel-Raouf M, Kamal RS, Hegazy DE, Sayed A (2023). Gamma irradiation synthesis of carboxymethyl chitosan-nanoclay hydrogel for the removal of Cr (VI) and Pb (II) from aqueous media. J. Inorgan. Organometal. Polym. Mater..

[22] Sayed A, Mazrouaa AM, Mohamed MG, Abdel-Raouf MES (2023). Green synthesis of chitosan/erythritol/graphene oxide composites for simultaneous removal of some toxic species from simulated solution. Environ. Sci. Pollut. Res..

[23] Reda M, Ateia EE, El-Dek SI, Arman MM (2024). New insights into optical properties, and applications of Zr-doped BaTiO_3_. Appl. Phys. A.

[24] Sayed A, Hany F, Abdel-Raouf MES, Mahmoud GA (2022). Gamma irradiation synthesis of pectin-based biohydrogels for removal of lead cations from simulated solutions. J. Polymer Res..

[25] Arman MM (2022). Structural, morphological and magnetic properties of hexaferrite BaCo_2_Fe_16_O_27_ nanoparticles and their efficient lead removal from water. Appl. Phys. A.

[26] Khoso WA, Haleem N, Baig MA, Jamal Y (2021). Synthesis, characterization and heavy metal removal efficiency of nickel ferrite nanoparticles (NFN’s). Sci. Rep..

[27] Coutinho PV, Cunha F, Barrozo P (2017). Structural, vibrational and magnetic properties of the orthoferrites LaFeO_3_ and YFeO_3_: A comparative study. Solid State Commun..

[28] Arman MM (2023). Effect of divalent cations (Co^2+^ and Ni^2+^) on microstructure, physical properties and application of Nd. J. Rare Earths..

[29] Arman MM (2023). Highly efficient lead removal from water by Nd_0.90_Ho_0.10_FeO_3_ nanoparticles and studying their optical and magnetic properties. Sci. Rep..

[30] Hasan AM, Keshawy M, Abdel-Raouf MES (2022). Atomic force microscopy investigation of smart superabsorbent hydrogels based on carboxymethyl guar gum: Surface topography and swelling properties. Mater. Chem. Phys..

[31] Arman MM (2023). The effect of the rare earth A-site cation on the structure, morphology, physical properties, and application of perovskite AFeO_3_. Mater. Chem. Phys..

[32] Rajaitha PM, Hajra S, Padhan AM, Panda S, Sahu M, Kim HJ (2022). An electrochemical sensor based on multiferroic NdFeO_3_ particles modified electrode for the detection of H_2_O_2_. J. Alloys Comp..

[33] Yuan SJ, Ren W, Hong F, Wang YB, Zhang JC, Bellaiche L, Cao G (2013). Spin switching and magnetization reversal in single-crystal NdFeO_3_. Phys. Rev. B.

[34] Vavra M, Zentková M, Mihalik M, Mihalik M, Lazúrová J, Girman V, Jaglicic Z (2017). Exchange bias effect in NdFeO_3_ system of nanoparticles. Acta Physica Polonica A.

[35] Ghaffar DN, Arman MM, El-Dek SI, Ramadan R (2024). Studying the preparation, characterization, and physical properties of NiFe_2_O_4_, TiO_2_, and NiFe_2_O_4_/TiO_2_ nanocomposite. Appl. Phys. A.

[36] Ateia EE, Gawad D, Mosry M, Arman MM (2023). Synthesis and functional properties of La_2_FeCrO_6_ based nanostructures. J. Inorgan. Organometal. Polymers Mater..

[37] Wasewar KL (2010). Adsorption of metals onto tea factory waste: A review. Int. J. Recent Res. Appl. Stud..

[38] Al-Senani GM, Al-Fawzan FF (2018). Adsorption study of heavy metal ions from aqueous solution by nanoparticle of wild herbs. Egypt. J. Aquatic Res..

